# Ethical Counseling on Assisted Suicide in German and Swiss Right‐To‐Die Organizations: Challenges and Perspectives

**DOI:** 10.1111/bioe.70092

**Published:** 2026-01-31

**Authors:** Dieter Birnbacher, Peter Schaber

**Affiliations:** ^1^ Department of Philosophy University of Düsseldorf Germany; ^2^ Department of Philosophy University of Zurich Switzerland

**Keywords:** assisted suicide, counseling, ethics committee, Germany, right‐to‐die organizations, Switzerland

## Abstract

During the last years, more and more countries have introduced a practice of assisted dying in their medical system and regulated it by separate laws or by additions to the existing body of criminal law. In this respect, the two neighboring countries, Germany and Switzerland, are exceptional cases. In both countries, there exists a long‐standing and largely uncontested legal prohibition of euthanasia, but only minimal state regulation concerning assisted suicide. Right‐to‐die organizations face the challenge of filling the gap by defining their own rules. In this, the largest right‐to‐die organizations in both countries are advised by individual ethicists or ethics committees, with the mission to uphold ethical standards. The paper compares the role of ethical counseling in these organizations (in which the authors are the heads of the respective ethics committees) and describes the scope of their counseling work and the extent of their responsibility for maintaining an ethically defensible practice.

## 1 Introduction: Germany and Switzerland—Black Swans

In the last years, there has been a gradual awakening concerning patient rights. Respecting patient autonomy has established itself as a leading principle not only of medical ethics but also of international guidelines such as the WMA International Code of Medical Ethics and, in many cases, of national professional law. This development has not been without repercussions on the theory and practice of self‐determined dying. More and more countries have liberalized their legal rules concerning stopping treatment at the patient's request, assisted suicide, and euthanasia, or are in the process of doing this, mostly in the democratic part of the world. These developments have been regularly accompanied by a good deal of controversy among politicians, lawyers, physicians, and other professionals, as well as religious leaders, interest groups, and society at large. As was to be expected, the solutions found differ in how they define the balance between the equally legitimate objectives of honoring patient wishes and protecting the vulnerable from self‐harming by irrational and uninformed death wishes. However diverse these solutions have turned out to be in content, they have in common that, in most cases, they have been fixed by state regulation and either integrated into the existing body of criminal law or established as separate laws.

In this respect, the two neighboring countries, Germany and Switzerland, are notable exceptions. In both countries, there exists a long‐standing and largely uncontested legal prohibition of euthanasia, but only minimal state regulation concerning assisted suicide. In July 2023, two different proposals for a law on assisted suicide failed to get a majority in the German *Bundestag*. Neither is there a regulation, in either country, of Voluntary Stopping Eating and Drinking as a form of self‐determined (and, in most cases, medically accompanied) dying. At the same time, the practice of assisted suicide is growing fast in both countries, though on very different levels. In both countries, the limits set by constitutional and criminal law are wide and leave room for varying solutions. They define the conditions under which assisted suicide may be practiced, but they leave open important details. They do not specify, for example, who is authorized to function as an assistant by providing the lethal drugs to the persons willing to end their lives, and they do not indicate the procedures to be followed. Both in Switzerland and present‐day Germany, state laws function as no more than a framework for autonomous rule‐setting by right‐to‐die organizations and medical associations.

### 1.1 The Legal Situation in Switzerland

As far as Switzerland is concerned, the legal limits are defined by two rules of criminal law. The first is that the person wishing to die and asking for assistance in voluntarily ending life must possess decision‐making capacity (*Urteilsfähigkeit*). As far as assistance in suicide is carried out with persons with no or limited decision‐making capacity, the assistant runs the risk of being persecuted for indirect manslaughter. The second is that assistance in suicide must not be carried out for “selfish motives”,^1^ for example, by suggesting suicide with his or her assistance to a relative with the view to inherit his or her fortune. This legal ruling, however, has never been applied so far, at least not in a case of assisted suicide. In June 2011, the Swiss *Bundesrat* decided not to introduce an explicit regulation of assisted suicide into Swiss criminal law.^2^

### 1.2 The Legal Situation in Germany

In Germany, the legal situation is defined by the judgment of the German Federal Constitutional Court in February 2020 that it is part of the personal right of every human being to decide freely when and how to end one's own life. It went even further and stated that every person with a serious, firm, and relatively constant wish to end his or her life should have a realistic chance to use the voluntary help of third parties for this purpose.^3^ It is a more or less unique feature of this ruling that, as a human right, this right is not limited to a particular condition of the person. The person need not be terminally ill or suffer from an incurable, painful disease. The only conditions the person who is willing to die has to meet are that he or she has the required decision‐making capacity, is fully informed about the alternatives, and has a considered and constant wish to die that is not due to manipulation or pressure from others. At the same time, the Court left it to the legislator to introduce legal rules to control the practice (without, however, formally requiring it), preferably differentiated according to the risks involved.

As in Canada and Austria, the background of this judgment was a complaint against the existing legal situation by organizations, physicians, and patient groups pleading that their constitutional right to personal choice regarding the time and manner of ending their lives or, respectively, their freedom to exercise their professional role as a physician was intolerably restricted by the existing regulation.

## 2 The Situation of Right‐To‐Die Organizations

In both countries, the legal situation is a challenge for right‐to‐die organizations. With the primary aim of acting as advocates of those who wish to end their lives and given the absence of provisions for the implementation of this right by the state, they see themselves forced to fill the gaps in regulation by defining their own rules, partly aligning with the rules proposed by their countries' medical associations.

Right‐to‐die organizations have existed in both countries for more than 40 years, with a slightly different focus. Whereas Swiss associations like EXIT Deutsche Schweiz and EXIT Suisse Romande took up assisted suicide from the very beginning, some of the German organizations focused on the legal recognition of advance directives, strengthening patient rights, and protecting patients against medical overtreatment. Only a few of them were involved in the practice of assisted suicide. Around 2000, one of the German right‐to‐die organizations, under the leadership of a former politician, started to draw the attention of the general public to their practice through actions of a more or less provocative nature, with the result that the conservative majority in the German medical establishment and parts of the political class felt the necessity to introduce legal restrictions. These came to be realized in 2011 with a prohibition of assisting suicide for physicians in professional law^4^ and in 2015 with a prohibition of assisting suicide on a regular basis by physicians and others in the Criminal Code.^5^

Whatever the factual situation, there is, in both countries, a certain proportion of citizens wanting to see the practice reliably regulated, given, on the one hand, the high degree of acceptance of the practice (under certain conditions), and, on the other, the undeniable risks of misuse and abuse. In Switzerland, the high social acceptance of right‐to‐die organizations became manifest in a vote on an initiative that was under discussion in the Canton of Zürich in 2011. The initiative, which called for a ban on right‐to‐die organizations, was rejected with 85% of the votes. Nevertheless, in Switzerland, political attempts to regulate the practice failed. The various parties involved could not agree on guidelines on how to regulate assisted suicide, and the various views on the matter differed too much. EXIT even favors the status quo because they fear that a legal regulation that the political parties and the various interest groups would agree on would be, from their point of view, too restrictive. In Germany, a high acceptance of assisted suicide as a form of self‐determined dying has consistently emerged from surveys throughout the last 20 years. Interest has recently been pushed by legal proceedings against two prominent protagonists, resulting in severe penal sanctions for indirect manslaughter. On the other hand, membership in the largest right‐to‐die organizations in both countries, EXIT Deutsche Schweiz in Switzerland (located in Zürich) and DGHS in Germany (located in Berlin), exploded. Membership of EXIT is presently at a peak of 185.000, making it one of the largest associations in German‐speaking Switzerland. The largest German right‐to‐die organization, DGHS, has at present 60.000 members, with a rate of growth of around 2.000 new members per month.

The fact that assisted suicide with the help of right‐to‐die organizations has a much longer history in Switzerland than in Germany may be one of the factors for the significant difference in the number of cases of assisted suicide in both countries (another factor may be the legendary Swiss love of freedom). Whereas in both countries the rate of non‐assisted suicide in recent years has been relatively constant (at about 1% of the total of all deaths) [1], the number of cases of assisted suicide in Switzerland is at present at 2.1% of all deaths [2], four times as high as that of 2010 [3]. In Germany, where assisted suicide is not statistically counted as a separate category, the number of cases of assisted suicide carried out or enabled by the three relevant organizations amounts at present to about 0.1% of all deaths. It can be expected to rise in the future.

## 3 Issues of Ethical Counseling in German and Swiss Right‐To‐Die Organizations

In both countries, the largest right‐to‐die organizations are advised by individual ethicists or by ethics committees with the mission to uphold ethical standards. The present authors are the heads of the respective ethics committees of EXIT and DGHS.^6^ These associations will serve, in the following, as exemplary cases.

A comparison of the role of ethicists and ethics committees in both associations shows that there is a significant overlap in the issues discussed. In both associations, ethicists and ethics committees advise the respective boards on matters of principle and in individual cases. There is, however, a clearer focus, in the German case, on rule‐setting and general strategy rather than on decisions in controversial individual cases.

One of the ethical issues of principle is, in both associations, whether the assistance in suicide carried out or enabled by the respective association should include tired‐of‐life cases in which there is no health problem but suffering from a condition of being tired of life, a condition in which the prospect of going on living is experienced as being doomed to a dreary existence lacking meaning and direction. A side aspect of this is the wish for a synchronous suicide of long‐married elderly couples, of which one partner is suffering from a grave disease and faces death in the near future, whereas the other faces the subjectively unbearable prospect of being left alone.

The proportion of cases of assisted suicide carried out with the help of right‐to‐die organizations not directly related to grave, incurable, or lethal diseases in Germany is small but significant. According to the DGHS White Book for the years 2020 and 2021, the great majority of cases were cases of severe illness, with an average age of 78 years [4]. Cases, in which the motives of the death wish were tiredness of life, or the wish not to be left alone by a dying partner, amounted to 15%. In Switzerland, cases of being tired of life and wishing not to be left alone after the voluntary death of a partner are not separately listed.

These cases are regularly a point of debate. In Switzerland, there is a disagreement between the Swiss Academy of Medical Sciences (SAMW) and the right‐to‐die organizations on the issue of whether a medical condition has to be satisfied to justify suicide assistance. The SAWM holds the view that such a medical condition is a necessary condition. In their most recent guidelines, they write:

To justify the fact that assisted suicide falls within the medical responsibility, there must be medically recognizable symptoms of illness or functional limitations present [5].

The point is that to assist someone in ending his or her life would only fall within the responsibility of a physician if there was a medical condition present. Healthcare professionals must be able to trace the suffering of a person who wants to end his or her life back to a disease or dysfunction. Only then, so the argument goes, can assisted suicide by healthcare professionals be justified.

It should be observed, however, that the notion of “functional limitation” is permissive enough to subsume at least the majority of cases accepted by the Swiss right‐to‐die organizations for reasons of tiredness of life. The wording can be interpreted as a verbal compromise rather than a restriction. After all, who among the elderly people asking for assisted suicide for reasons of high age can be expected to be without multimorbidity?

Furthermore, the conditions set by the SAMW are not regarded as part of the general Swiss law by the authorities. This was made clear in a recent legal case, in which the former president of EXIT Suisse Romande, Pierre Beck, was acquitted of manslaughter. The directives if the SAMW were stated to be valid only for the members of the Swiss medical association FMH (Foederatio Medicorum Helveticorum) whose rules the SAMW took over in 2022 and in which membership is voluntary. Beck had helped the wife of a severely ill man to die, together with her husband, though she herself was in good health.

Another issue in which ethical bodies have been consulted is the question of whether the suicide wish of a person asking for an assisted death must meet, over and above the standard criteria of having decision‐making capacity, being voluntary, well‐considered, stable over time, and not made under pressure, the criterion of being plausible to others (“Nachvollziehbarkeit”) [6]. This criterion implies that the death wish should be intelligible to others. This condition is not part of the standard criteria. Even if a death wish is well‐informed and not the symptom of a mental disease or disturbance, it is sometimes beyond the scope of rational comprehension. Wishes to get access to assisted suicide in cases of this kind are regularly turned down by the relevant organizations.^7^

In Switzerland, the right‐to‐die organizations favor the view that the death wish need not arise from a medical condition. They argue that assisted suicide can also be justified if the death wish is due to existential suffering independently of a medical condition. For them, subjectively intolerable suffering is the relevant condition, regardless of whether the person suffers from an incurable disease. They hold, however, that the wish of the person who wants to end her life has to be intelligible to those who assist.

The EXIT working group “Assisted Suicide with Old Age People” goes further, arguing that in the case of elderly people, the death wish does not even have to be understandable to the potential assistant. In their view, it is enough that the old person has decision‐making capacity and the death wish is autonomous. They argue that a requirement of plausibility would be overly paternalistic. Younger people might be insufficiently able to empathize with very old people to understand why they want to end their lives. They think that it may not be acceptable to very old people that a third party claims to know better what is good for the person who wishes to die [7].

In response to this statement of the working group, the EXIT's ethics committee came up with the following statement regarding assisted suicide with elderly people:

“Closer examination makes it clear that old age is not an additional criterion for the permissibility of assisted suicide. Therefore, the discussion about so‐called “old‐age suicide” is about nothing other than rethinking the medical and non‐medical criteria for the permissibility of assisted suicide in general. However, in old age, it is much more likely that the criteria for authorized assisted suicide are met (see below). It is important to note that permissibility must also be assessed from the perspective of the person assisting the dying (carer, doctor). In our view, the following criteria are important from the perspective of the person willing to die and the person assisting the dying: From the perspective of the person who wants to end her life, there must be (a) subjectively unbearable suffering due to symptoms of illness and/or functional limitations, psychosocial aspects, existential suffering, or a poor prognosis. The suffering situation must also (b) be irreversible, and the wish to die must (c) not merely be the result of acute symptoms but must result from a well‐considered assessment of one's life situation. And from the perspective of the assisted dying person: The wish to die must be comprehensible to the assisted dying person and the person wishing to die must need help to access a gentle and safe method of suicide”.^8^

In this case, the primary motivation of the Ethics Committee was the wish that the organization should not sacrifice the thrust and unity of its mission to the ambition to satisfy the wishes of all and each of their members for ultimate certainty to be assisted at the end of their lives. Apart from that, the wish to integrate different views, mentalities, and temperaments under one leading idea is, in these associations, no less psychologically costly for the integrators than it is in other associations with highly emotional issues. Following the dispute, the members of the Ethics Committee were confronted with a good deal of personal criticism.

The situation is similar in Germany, where a certain proportion of members is in favor of the Benelux framework that enables people to determine in an advance directive to get euthanasia in a state of advanced dementia, when assisted suicide will not be possible because the requirement of full decisional capacity can no longer be met. In contrast to this, the Ethics Committees of the German associations, though expressing understanding for the strong wish of many members to make provisions for the case of dementia (which, for many, is a more serious threat than severe somatic illness), have moral objections against extending the scope of advance directives with the consequence of revisions of the criminal code. Assisted suicide means that a threshold lies between patient wishes and death, which he or she has to overcome by his or her effort. It puts the voluntariness and responsibility of death to a more severe test than euthanasia, which is more likely to be interpreted as a medical procedure in which the patient is passive rather than active and in which he or she receives death from the hands of physicians in the same way as normal medical treatment. Though the “normalization” of self‐determined dying feared my many German conservatives seems inevitable in the long run, it seems doubtful whether death should be normalized to such a degree that it loses its exceptional character as the final and irreversible end of all conscious experience.

In general, ethicists and ethics committees are consulted by the governing boards of their associations primarily to set up the guidelines defining the procedures and standards for everyday practice. As a rule, right‐to‐die organizations are open to ethical advice, not least for strategic reasons. They have a vital interest in keeping up high standards to safeguard their reputation in a controversial and emotionalized public debate. In addition, the guidelines set up by right‐to‐die associations inevitably stand in competition with similar guidelines set up by medical associations, such as, in Switzerland, those of the SAMW, the Academy of Medical Science, or, in Germany, with the recommendations of the Deutsche Gesellschaft für Schmerzmedizin (German Society for Pain Medicine) [8]. All guidelines state that the four‐eyes‐principle has to be followed in all decisions to offer assistance, and partly also throughout the process. However, this principle is open to more than one interpretation and does not preclude dissent over how it should be translated into procedures. For reasons of practicability and cost efficiency, DGHS and EXIT require the participation of only one physician (except in cases of psychiatric disease), whereas others require the participation of at least two physicians to reduce potential bias.

Another guideline decision concerns the provisions for death wishes in cases of psychiatric illness. In such cases, a psychiatrist or psychotherapist must be consulted to clarify how far the death wish is a symptom of the psychiatric disease and how far it results from a reflected judgment on whether the psychiatric disease and its symptoms are subjectively unbearable. In the first case, assistance would be contrary to the legal demand of full voluntariness and must be rejected, however deplorable the situation is for the concerned individual who is left to his or her fate [9]. In the second case, assisted suicide is an option, though only on the condition that a psychiatrist (or a clinical psychologist) states that the candidate has sufficient decision‐making capacity.

Since it is part of the commitment of most right‐to‐die organizations to lobby for the (further) liberalization of aid in dying, ethicists in a counseling role are also confronted with strategic questions as to the line to be taken by the lobbying messages. One of these questions is whether there should be state regulation of assisted suicide at all. Are the limits defined by the criminal law sufficient to exclude misuse and abuse? This issue, again, is more complex than it appears at first sight and might profit from ethical advice no less than the setting up of guidelines. There are obvious pros and cons. On the side of pros, there is the fact that even a liberal legal framework does not guarantee that people able to offer professional help with suicide are prepared to offer it. One of the reasons to be reluctant is a lack of orientation about the form and the limits of how to go about it. In Germany, even among physicians, there are still considerable uncertainties about the extent to which the practice is legal according to general law or compatible with the professional code. In this respect, the widespread criticism of conservative circles, according to which the practice is at present operating in a “gray area” without clear directions about what is permissible and what is not, is not completely unfounded. This situation is doubly unsatisfactory because of the growing interest in assisted suicide on the part of the general public, not least as a consequence of increased public discussion and a growing presence of the topic in press reports, film, and TV productions. Lack of orientation on the part of physicians motivates some of them to refuse to help and leads to a good deal of helplessness on the part of interested patients looking for opportunities to exercise their basic right to a self‐determined death, as stated by the Constitutional Court. They feel betrayed by politicians who compete with one another in urging the need for regulation in order to protect the vulnerable and tend to overlook the need of others (the majority) to see their basic right to a self‐determined death realized by practical solutions.

The fact that one of the main medical authorities in Germany, the German *Bundesärztekammer*, will very likely not come up with concrete guidelines for physicians as long as they do not know what the state legislation will look like, is seen as a further reason to legally regulate matters of assisted suicide. Medical practitioners are particularly unhappy about the lack of legal regulation. For this reason, several medical associations (such as the German Association of Medical Practitioners) have announced that they will publish their own rules.^9^

There are, on the other hand, weighty objections against the proposed legal regulations. It is argued that those that were proposed so far have a tendency to bureaucratism, hardly compatible with the personal and intimate nature of the act. They are rejected by many patients and physicians as excessively restricting autonomy. What is seen as particularly problematic is that they imitate the existing (though highly controversial) control mechanisms in the context of abortion and thereby pay insufficient attention to the categorical difference between voluntarily and autonomously ending one's own life and ending the lives of others. Other proposals impose prohibitively high burdens, for example, by demanding the involvement of psychiatrists and psychotherapists in all cases, irrespective of the plausibility of the underlying death wish, thereby severely limiting access to assisted suicide, given the existing scarcity of psychiatrists and psychotherapists and long waiting times. Accessibility for everyone, regardless of educational and social status, is, however, an aim shared by all relevant organizations.

A further issue in both countries concerns the extent to which physicians should be involved in assisted suicide and whether assisted suicide should be completely ‘de‐medicalized’. This is not a legal requirement in either Germany or Switzerland. However, it remains an ethical question as to who should be involved in assisted suicide. This question is as urgent as it is fundamental. Though a small minority of physicians are regularly involved in both countries, the majority of physicians, especially specialists in palliative care and geriatrics, who are in particularly close contact with gravely ill older people, are mostly reluctant to get involved. As emerges from a recent Swiss study, only a third of the palliative care physicians surveyed see assisted suicide as an actual or potential instrument of palliative care. One‐third expressed ambivalence, citing possible stigmatization within the profession or clinic and a lack of expertise, and another third disapproved [10]. Nevertheless, at present, the chances to get assistance are significantly worse in Germany than in Switzerland, especially for persons with a death wish who have no family doctor or for whom physicians are not required to care because they are not one of their patients.^10^

## 4 Ethical Counseling Regarding Assisted Suicide—Ethical Advice and Conceptual Analysis

The main task of ethicists and ethics committees in right‐to‐die organizations is to give advice on normative matters and counsel the governing board on which rules should be followed in practice, and sometimes also on how controversial cases should be dealt with. In the case of Germany, the recipients of this kind of advice are the persons constituting the governing body. There is, in this process, no direct interaction with the people carrying out the assistance or with the applicants. Nor are the materials the applicants hand in to motivate their application and provide information about their medical and social background accessible to the ethicists, except in the cases in which there is a controversy about admission or rejection rooted in ethical dissent. For privacy reasons, these materials are circulated only in the team of psychologists and lawyers deciding on admission or rejection and in the individual team of the physician and the lawyer carrying out the assistance. In Switzerland, the assistants handing over the lethal drug are often present in the conversations preceding assisted dying. It is up to them to decide whether a physician should be asked to prescribe the lethal drug. The last word is the physician's. There will be no assisted suicide if no physician can be found who is prepared to make the prescription.

Which direction does the advice given by ethicists and ethics committees take in normative respects? As a rule, they recognize as their guiding principle the attempt to strike a balance between the aims of making the opportunity for a self‐determined death available to as many people as possible, irrespective of social background, and of excluding as far as possible misuse and abuse. It goes without saying that in these considerations, ethicists are not completely neutral. They would not be involved if they had no sympathies for supporting the practice of assisted suicide. Their view is, however, always only one of many views expressed in the discussions leading to judgment on the introduction or change of internal guidelines and the policies of admitting or rejecting controversial applications for assisted suicide. The last word is always on the part of the board elected by the members of the association or their elected delegates. Nevertheless, the influence of ethicists or ethics committees is, as a rule, fairly strong.

The fact that the ethical views are not decisive for the board is partly due to the fact that they compete with strategic considerations. After all, organizations like EXIT and DIGNITAS have, besides their roles as providers of advance directives and of opportunities for assisted suicide, a political role as players in the field of public debate, advocating more liberal attitudes and practices concerning self‐determined death. Strategic considerations often overlap with ethical considerations, but this is not always the case. Sometimes, the ethical considerations go further than what seems politically appropriate to postulate in public; sometimes, they are more conservative than what the majority of citizens think is justified (such as the practice of euthanasia on the model of Canada and the Netherlands). Given the separateness of ethical and strategic considerations, it is one of the challenges of ethics committees to maintain their independence. Strategic considerations are not properly their business, though ethical and strategic issues often get intermingled, especially with ethicists or committee members who act at the same time as functionaries. These members, then, may have a role conflict. On the other hand, they may be indispensable as mediators between the ethics committee and the executive bodies of the organization, enabling them to keep close contact and to facilitate communication between them. In other respects, the members of Ethics Committees are free to bring up all moral aspects of dealing with death wishes they think relevant. There is ample room in these bodies for a multiplicity of voices and perspectives.

### 4.1 Analytical and Conceptual Aspects

Another task ethicists are often expected to fulfill in associations with a primarily pragmatic agenda lies outside the sphere of normative considerations and concerns analytical and conceptual issues. Ethicists and the members of ethics committees are mostly academics trained in analytical thinking. Experience shows that normative and conceptual questions are intertwined and that communication to the general public on positions and decisions taken depends heavily on conceptual clarity. This function is especially important in the area of medically assisted dying because misunderstandings and confusion abound in both the general public and the medical community. Surveys regularly show, for example, that many physicians think that actively withdrawing treatment (as by pressing the button of a respirator or stopping a cardiac pacemaker) constitutes active killing and is (in the two countries in question) punishable as euthanasia. Apart from that, normative assessments quite often depend on conceptual schemata, such as in the case of stopping eating and drinking which is seen by many as a form of (assisted) suicide and therefore rejected on grounds of conventional or religious morality, whereas for others it is a form of withdrawal of treatment on the patient's request and tolerated or even seen as an obligation.

More relevant conceptual questions surrounding assisted suicide arise in relation to legally binding access criteria, including concepts such as “decision‐making capacity” and “voluntariness”. How these concepts are interpreted in concrete cases is crucial for their legal and ethical permissibility. This holds especially for cases in which the wish for assisted suicide comes from persons with a mental disease, such as chronic deep depression, and where the death wish is just one of the symptoms of the disease. Such cases raise the question of how to distinguish between a death wish as a symptom of the mental disease in its acute phase and a death wish as a result of careful reflection during a remission period. The analysis of concepts like “voluntariness” and “decision‐making capacity” is also important because there are disagreements about conceptual issues. Opponents of assisted suicide sometimes hold the view that death wishes are not just involuntary when they are caused by psychosis and misinformation, but also when they are influenced by socio‐cultural factors such as the ideological dominance of ideals of self‐realization or hedonistic ideals of a life without suffering. Advocates of assisted suicide, on the other hand, sometimes take it to be the case that death wishes are voluntary whenever the person who has this wish is not mentally deranged, and the wish is intelligible.^11^ Thus, both advocates and opponents of assisted suicide often interpret the concept of voluntariness differently, underscoring the need for a careful conceptual analysis of terms such as “voluntariness”.

## 5 The Question of Responsibility

Finally, there arises the question of the responsibility of ethicists and ethics committees. Here, as in other contexts, it is useful to distinguish between legal and moral responsibility. The legal responsibility of offering opportunities for assisted suicide lies outside the sphere of counseling ethicists. In this respect, ethicists and ethics committees are in the same situation as ethics committees in hospitals or nursing homes. Legal responsibility for admitting or rejecting applications for assisted suicide lies in the DGHS, with the team of psychologists and lawyers deciding whether to hand over the application to the executive teams of lawyers and physicians or not. These, in turn, are legally responsible for accepting or not accepting the applications filtered out by the psychologists, after at least two interviews with the applicants. The ultimate responsibility then lies with the physician carrying out the assistance. With EXIT, the distribution of responsibility is slightly different. The legal responsibility for assisting someone to end his or her life lies only with the physician prescribing the lethal drug. The members of the right‐to‐die organizations who assist someone in ending her life are arguably morally but not legally responsible for their assistance. Similarly, ethicists and ethics committees never have the last word in decisions concerning statutes and, thus, concerning the extent of the kind of help the organization is prepared to offer.

This does not imply that the people involved in ethical counseling are exempt from legal and moral responsibility. Though their advice is not decisive, it may have an important indirect part both in setting up the rules and in evaluating individual applications. The extent to which they are morally responsible for possible misuse equals the extent of their contribution. In Germany and Switzerland, they can also be held legally responsible for negligent homicide if they provide incorrect advice.

## Endnotes

1 Art 115 StGB.

2 Cf. Bericht des Bundesrates, Bern 2011.

3 Bundesverfassungsgericht: Urteil des Zweiten Senats, in Kraft getreten am 10. Dezember 2015, wurde am 26. Februar 2020 aufgehoben. http://www.bverfg.de/e/rs20200226_2bvr234715.html.

4 114. Deutscher Ärztetag Kiel 2011. Beschlussprotokoll, 176.

5 § 217 StGB (Geschäftsmäßige Förderung der Selbsttötung), in force from 10. 12. 2015 till 26. 2. 2020.

6 The Ethics Committee of EXIT currently consists of five members (two physicians, one psychologist, one philosopher, and one theologian). The Ethics Committee of the DGHS is currently about to be Restructured.

7 A recent case is that of a young homosexual with a desperate but unrealizable wish to found a “normal“ family (cf. Frankfurter Allgemeine Sonntagszeitung 17. 9. 2023, 12. The man killed himself after having been rejected.

8 EXIT, Internal Statement 2018, trans. PS.

9 Cf. Frankfurter Allgemeine Zeitung, 28.12. 2023, 10, Deutsche Gesellschaft für Allgemeinmedizin und Familienmedizin e.V. Der Umgang mit dem Wunsch nach Suizidassistenz in Hausärztlichen Praxen. DEGAM S1‐Handlungsempfehlung 2024.

10 It has to be noted here that the German Federal Constitutional Court has declared the monopoly of doctors as unlawful. Bundesverfassungsgericht, February 26, 2020.

11 Cf. Schöne‐Seifert, Bettina, Beim Sterben Helfen—Dürfen Wir Das? 2020. J.B. Metzler‐Verlag.
